# Phylogenetics and Differentiation of *Salmonella* Newport Lineages by Whole Genome Sequencing

**DOI:** 10.1371/journal.pone.0055687

**Published:** 2013-02-11

**Authors:** Guojie Cao, Jianghong Meng, Errol Strain, Robert Stones, James Pettengill, Shaohua Zhao, Patrick McDermott, Eric Brown, Marc Allard

**Affiliations:** 1 Department of Nutrition and Food Science, University of Maryland, College Park, Maryland, United States of America; 2 Biostatistics Branch, Center for Food Safety and Applied Nutrition, U.S. Food and Drug Administration, College Park, Maryland, United States of America; 3 Food and Environment Research Agency, York, United Kingdom; 4 Division of Microbiology, Office of Regular Science, Center for Food Safety and Applied Nutrition, U.S. Food and Drug Administration, College Park, Maryland, United States of America; 5 Division of Animal and Food Microbiology, Office of Research, Center for Veterinary Medicine, U.S. Food and Drug Administration, Laurel, Maryland, United States of America; Columbia University, United States of America

## Abstract

*Salmonella* Newport has ranked in the top three *Salmonella* serotypes associated with foodborne outbreaks from 1995 to 2011 in the United States. In the current study, we selected 26 *S*. Newport strains isolated from diverse sources and geographic locations and then conducted 454 shotgun pyrosequencing procedures to obtain 16–24 × coverage of high quality draft genomes for each strain. Comparative genomic analysis of 28 *S*. Newport strains (including 2 reference genomes) and 15 outgroup genomes identified more than 140,000 informative SNPs. A resulting phylogenetic tree consisted of four sublineages and indicated that *S*. Newport had a clear geographic structure. Strains from Asia were divergent from those from the Americas. Our findings demonstrated that analysis using whole genome sequencing data resulted in a more accurate picture of phylogeny compared to that using single genes or small sets of genes. We selected loci around the *mutS* gene of *S*. Newport to differentiate distinct lineages, including those between *invH* and *mutS* genes at the 3′ end of *Salmonella* Pathogenicity Island 1 (SPI-1), *ste* fimbrial operon, and Clustered, Regularly Interspaced, Short Palindromic Repeats (CRISPR) associated-proteins (*cas*). These genes in the outgroup genomes held high similarity with either *S*. Newport Lineage II or III at the same loci. *S*. Newport Lineages II and III have different evolutionary histories in this region and our data demonstrated genetic flow and homologous recombination events around *mutS*. The findings suggested that *S*. Newport Lineages II and III diverged early in the serotype evolution and have evolved largely independently. Moreover, we identified genes that could delineate sublineages within the phylogenetic tree and that could be used as potential biomarkers for trace-back investigations during outbreaks. Thus, whole genome sequencing data enabled us to better understand the genetic background of pathogenicity and evolutionary history of *S*. Newport and also provided additional markers for epidemiological response.

## Introduction

Salmonellosis is a major contributor to global public health burden. In the United States, non-typhoid *Salmonella* annually cause an estimated 1.4 million gastroenteritis cases [1] and several billion dollars of economic loss [2]. Non-typhoid *Salmonella* account for only 11% of foodborne illnesses [3], but cause 35% of hospitalizations and 28% of the deaths related to foodborne illnesses [4]. There are over 1,500 serotypes in *Salmonella. enterica* subsp. *enterica* [5]. According to CDC [3], *S.* Newport ranked in the top three *Salmonella* serotypes associated with foodborne outbreaks in the United States. The number of *S.* Newport outbreaks increased markedly since 1995, causing at least 100,000 infections annually [3]. *S*. Newport was responsible for several major outbreaks associated with tomatoes, ground beef, alfalfa sprouts, and other food products since 2002 [3], [6], [7], [8], [9]. *S*. Newport displays high levels of genomic diversity and is polyphyletic according to multilocus enzyme electrophoresis (MLEE) [10] and multilocus sequence typing (MLST) [11], [12], [13]. *S*. Newport has been split into three lineages in its evolutionary tree using MLST [14]. Most strains from Europe belong to *S*. Newport Lineage I, whereas most strains from North America belong to *S*. Newport Lineages II and III [14].

Several studies [15], [16] suggested that recombination events played a key role in the evolution of *Salmonella*. Brown et al. [17] indicated that *mutS* evolution in *Salmonella* genomes was distinct from the whole genome, and recombination events were not rare in the loci around *mutS*, which includes 3′ end region of SPI-1, *ste* fimbrial operon and *cas*. SPI-1 is a 40 kb gene cluster encoding Type III Secretion System (T3SS) [18]. It was identified in both *Salmonella enterica* and *Salmonella bongori*, although one study reported that an *S*. Senftenberg clinical strain did not have the SPI-1 [19]. CRISPRs/*cas* are present in most archaea and approximately 40% of bacteria [20], [21] and thought to be an important immune system to protect bacteria against foreign genetic elements as well as to help microbes survive phage predation; the CRISPR/cas system also facilitated the microbes to adapt to specific niche [22], [23], [24]. A recent study on CRISPR/*cas* system in *E. coli* and *Salmonella* revealed phylogeny of the *cas* protein family for different serotypes [25].

Since the first two *Salmonella* whole genome sequences were available in 2001 [26], , there were 28 complete genomes and over 90 draft genomes available in GenBank, including two *S*. Newport genomes, *S*. Newport SL254 and *S*. Newport SL317. Whole genome sequencing has been increasingly used as a tool for evolutionary studies and epidemiological investigations [9], [28], [29], [30], [31], [32]. In the current study, we performed pyrosequencing to obtain 16–24 × coverage (except strain from canine_AZ_2003 with 9 × coverage) of high quality draft genomes of 26 *S*. Newport strains from wide range of sources and geographic locations. Our data demonstrated the phylogenetic relationship among *S*. Newport strains and revealed variations around *mutS* gene, providing genetic evidence of recombination events. Moreover, genes that delineate major lineages and sublineages were identified and could be used as biomarkers to develop tools for trace-back studies for epidemiology and outbreak investigations.

## Materials and Methods

### Bacterial Strains

We selected 26 *S.* Newport strains isolated from diverse sources and geographic locations (Table 1). *S*. Newport SL254 (ABEN01000000) and *S.* Newport SL317 (ABEW00000000) were downloaded from GenBank as reference genomes. There are total 15 *Salmonella* genomes were chosen to be outgroup genomes according to pervious study [33], [34]. They are *S*. I 4, [5],12:i- SL474 (ABAO00000000), *S*. Kentucky CDC191 (ABEI00000000), *S*. Kentucky CVM29188 (ABAK00000000), *S*. Dublin CT_02021853 (CP001144), *S*. Gallinarum 287/91 (AM933171), *S*. Tennessee CDC07-0191 (ACBF00000000), *S*. Typhimurium 14028S (NC_016856.1), *S*. Typhimurium LT2 (NC_003197.1), *S*. Typhimurium SL1344 (NC_016810.1), *S*. Typhimurium D23580 (NC_016854), *S.* Choleraesuis SC-B67 (AE017220), *S.* Paratyphi C RKS4594 (CP000857), *S.* Virchow SL491 (ABFH00000000), *S*. Saintpaul SARA29 (ABAN00000000) and *S*. Hadar RI_05P066 (ABFG00000000).

**Table 1:** Characteristics of *Salmonella* Newport strains used in the study.

ID	Tree Label	PFGE Pattern Number	Antimicrobial Resistance Profile*	WGS Accession Number	Draft Genome Size (Mbp)	Number of Contigs
180	bison_TN_2004	JJPX01.0218	SUL	AHTJ00000000	4.71	95
181	caprine_TN_2004	JJPX01.0381	SUL	AHTK00000000	4.75	72
182	chicken_MO	JJPX01.0030	NA	AHTL00000000	4.71	71
183	ground_turkey_MD_2003	JJPX01.0502	NA	AHTM00000000	4.80	88
184	equine_TN_2004_1	JJPX01.0025	SUL	AHTN00000000	4.71	66
185	turkey_CO	NA	NA	AHTO00000000	4.74	64
186	frog_Vietnam	JJPX01.3333	NA	AHTP00000000	4.67	59
187	fish_Hong_Kong	JJPX01.0327	TET	AHTQ00000000	4.70	76
188	fish_Vietnam	JJPX01.1947	NA	AHTR00000000	4.67	53
189	equine_TN_2004_2	NA	SUL	AHTS00000000	4.96	72
190	swine_TX	NA	NA	AHTT00000000	4.92	73
191	cattle_NC_2003	JJPX01.0042	AMC,AMP,FOX,CHL,KAN,STR,SUL,TET,TIO	AHTU00000000	4.90	72
192	chicken_GA	JJPX01.0238	NA	AHTV00000000	4.93	70
193	cattle_AZ_2003	JJPX01.0014	AMC,AMP,FOX,CHL,STR,SUL,TET,TIO	AHTW00000000	4.93	69
194	canine_AZ_2003	JJPX01.0014	AMC,AMP,FOX,CHL,STR,SUL,TET,TIO	AHTX00000000	5.02	384
195	ground_turkey_NM_2008	JJPX01.0238	TET	AHTY00000000	4.93	85
196	ground_beef_GA_2004	JJPX01.0042	AMC,AMP,FOX,AXO,CHL,STR,SUL,TET,TIO	AHTZ00000000	4.89	77
197	swine_IL_2001	NA	AMC,AMP,FOX,CHL,GEN,KAN,STR,SUL,TET,TIO	AHUA00000000	4.69	44
198	shrimp_India	NA	NA	AHUB00000000	4.81	70
199	spinach_CO_2008	JJPX01.0538	NA	AHUC00000000	4.80	49
200	cheese_Mexico	JJPX01.0372	NA	AHUD00000000	4.65	74
201	squid_Vietnam	NA	NA	AHUE00000000	4.73	84
202	pepper_Vietnam	NA	NA	AHUF00000000	4.65	70
203	pig_ear_CA	NA	NA	AHUG00000000	4.73	62
117	farm_1_VA_2007#	NA	NA	AJMN00000000	4.81	91
118	farm_15_VA_2007#	NA	NA	AJMO00000000	4.81	75
NA	*S*. Newport SL254	NA	AMP, CHL, GEN, STR,AXO,SUL,TET	ABEN01000000	4.83	0
NA	*S*. Newport SL317	NA	NA	ABEW00000000	4.95	63

* AMC = Amoxicillin/Clavulanic Acid, AMP = Ampicillin, FOX = Cefoxitin, AXO = Ceftriaxone, CHL = Chloramphenicol, GEN = Gentamicin, KAN = Kanamycin, STR = Streptomycin, SUL = Sulfamethoxazole or Sulfisoxazole, TET = Tetracycline, TIO = Ceftiofur.

# These two samples were received from Eastern Shore of Virginia in 2007. Isolates may have been collected earlier than 2007.

### Pulsed Field Gel Electrophoresis (PFGE)

PFGE was performed according to the procedure as previously described [11].

### Genome Sequencing, Assembling and Annotation

Bacterial cells were pelleted from one ml of pure Tryptic-Soytone-Broth from overnight culture by centrifugation and DNA prepared using the DNeasy Blood & Tissue Kit (Qiagen, Valencia, CA) according to the manufacturer’s instructions. We sequenced 26 *S*. Newport strains using Roche 454 GS-FLX Titanium sequencer (Roche, Branford, CT) to obtain 16–24 × coverage of draft genomes (except strain from canine_AZ_2003 with 9 × coverage). This platform provides longer read lengths than other sequencing platforms to obtain raw sequences. *De novo* assemblies were performed using the Roche Newbler (v 2.3) software package. Annotation of resulting contigs was finished by NCBI according to Prokaryotic Genomes Automatic Annotation Pipeline (PGAAP) [35]. Phylogenetically informative SNPs were identified via two independent alignment methods: 1) multiple genome alignment of whole genome sequencing contigs using MAUVE [36], and 2) clustering of annotated open reading frames (ORFs) using reciprocal best Basic Local Alignment Search Tool (BLAST, http://blast.ncbi.nlm.nih.gov/Blast.cgi) hits with a 70% sequence identity setting followed by alignment with Multiple Sequence Comparison by Log-Expectation (MULCLE) [37].

### Phylogenetic Tree Reconstruction

Parsimony phylogenetic tree was constructed based on 147,780 concatenated informative SNPs by TNT [38] with finding minimum tree length 20 times and 100,000 iterations. We extracted seven housekeeping genes to perform MLST analysis. Concatenated housekeeping gene sequences were analyzed by TNT [38] with finding minimum tree length 20 times and 100,000 iterations. Moreover, we performed multiple sequence alignment using MULCLE [37] in SeaView 4 [39] and collected concatenated sequences of *cas* genes (*cas1*, *cas2*, *cas5*, *cse1*, *cse2*, *cse3* and *cse4*) with around 6k bps. Strains from frog_Vietnam, fish_Hong_Kong, fish_Vietnam, canine_AZ_2003 and pig_ear_CA were not involved in this analysis. We performed TNT [38] with finding minimum tree length 20 times and 100,000 iterations to display evolutionary relatedness of *cas* genes.

### Recombination Analysis

We used ClonalFrame [40] to analyze effects of recombination events on the evolutionary history of *S*. Newport Lineages II and III. *S*. DublinCT_02021853 was used as an outgroup genome to display the recombination events and substitutions between *S*. Newport Lineages II and III, which showed close relatedness to both lineages. All 29 *Salmonella* genomes were aligned using progressive MAUVE [36] with the default settings. We used the stripSubsetLCBs (locally collinear blocks) (http://gel.ahabs.wisc.edu/mauve/snapshots/) script to extract core blocks, which created core alignments longer than 500 bp that included all 29 genomes. We obtained total 510 LCBs. Given the computational demands necessary to analyze all 510 blocks simultaneously, we created three separate datasets each consisting of 50 randomly selected blocks. We ran ClonalFrame [40] on each of these three datasets with estimated parameters based on 200,000 generations of which the first 100,000 generations served as burn-in. The thinning interval was set to 100. We then used the Gelmin-Rubin statistic to determine whether the independent runs had converged on similar parameter estimates, which also provided evidence that random subsets of the genome did not bias our results. Furthermore, we used MAUVE [36] to compare the genomic organizations.

### Differences of Gene Cluster between *invH* and *mutS* Genes

We performed blastp to search best match of genes between *invH* and *mutS* genes, including Gene Clusters 1, 2 and 3. Tblastn was employed to verify the searching results.

### Pairwise Distance Matrix

MEGA 5.05 [41] was employed to construct evolutionary distance (no. of differences) over sequence pairs between groups with 1,000 bootstrap iterations.

### Searching for Most Variable Genes

Custom software was employed to look for the genes and informative SNPs to define the major lineages and sublineages. This was a GUI shell around open source software. In this analysis, we include 29 genomes including all 28 *S*. Newport strains and *S*. Choleraesuis SC-B67 as an outgroup genome. UClust algorithm [42] was employed to search gene families, using default settings with a 95% sequence identities cutoff. Maximum and minimum length of a gene cluster to search was 58,000 and 10 bp, respectively. MUSCLE [37] was employed to perform alignment with default settings. SNPs of these gene clusters were detected and were used to create phylogenetic matrix to construct phylogenetic tree using TNT [38] and count the informative SNPs that delineating major and sublineages on the nodes. Then we selected the genes containing the largest number of informative SNPs that defined major lineages and sublineages.

## Results

### Phylogenetic Relationship

Pyrosequencing was used to obtain 16–24 × coverage (except strain from canine_AZ_2003 with 9 × coverage) of high quality draft genomes of the 26 *S*. Newport strains with genome sizes ranging from 4.6 M bp to 5.0 M bp (Table 1). A total of 15 GenBank strains were selected as outgroup genomes to determine evolutionary relatedness and test polyphyly with *S*. Newport according to previous studies [33], [34] and one unpublished study of Center for Food Safety and Applied Nutrition, FDA. The outgroup genomes had close relatedness with *S*. Newport or were able to separate *S*. Newport strains. *S*. Newport SL254 and *S*. Newport SL317 were selected as reference genomes of *S*. Newport Lineages II and III, respectively [14]. *S.* Newport strains farm_1_VA_2007 and farm_15_VA_2007 are environmental isolates from a farm on the Virginia Eastern Shore. Among the 26 draft genomes, the largest genome size was 5.01 M bp of cannine_AZ_2003, while the smallest one was 4.65 M bp of pepper_Vietnam. There was no correlation between genome size and major lineages or sublineages.

**Figure 1:**
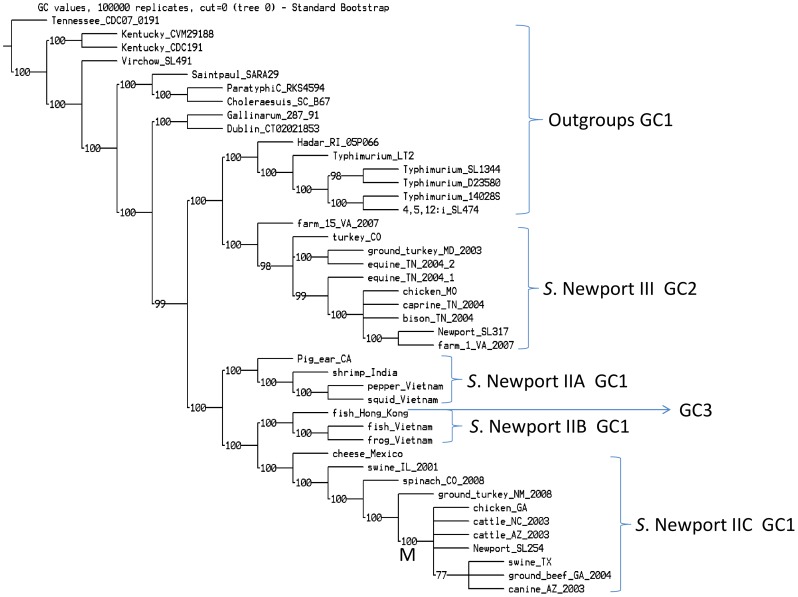
Parsimony phylogenetic tree of *S*. Newport and outgroup genomes. This phylogenetic tree was reconstructed by TNT [38] with 100,000 iterations based on 147,780 genome wide SNPs. All *S*. Newport strains were grouped into two major clusters, *S*. Newport Lineages II and III. Lineage II was further grouped into three sublineages, IIA, IIB and IIC. *S*. Newport displayed clear geographic structure. Asian strains were grouped together and divergent from ones from Americas. At the locus between *invH* and *mutS* genes, Lineage II and all outgroup genomes shared Gene Cluster 1; however, Lineage III strains shared Gene Cluster 2. Gene Cluster 3 was only found in strain from fish_Hong_Kong at the 3′ end of Gene Cluster 1. GC1 = Gene Cluster 1; GC2 = Gene Cluster 2; GC3 = Gene Cluster 3. Additionally, Node M includes most MDR strains in the current study.

A total of 147,780 informative SNPs were obtained from multiple genome alignment and were used to construct a parsimony phylogenetic tree (Figure 1) with 100,000 iterations by TNT [38]. All 28 *S*. Newport genomes (including *S*. Newport SL254 and SL317) were grouped into two major lineages (Figure 1), *S*. Newport Lineages II and III [14]. *S*. Newport Lineage II was further divided into sublineages IIA, IIB and IIC. Our data demonstrated that *S*. Newport displayed a clear geographic structure. For example, isolates from frog_Vietnam, fish_Hong_Kong, fish_Vietnam, shrimp_India, squid_Vietnam and pepper_Vietnam were placed in two sublineages (IIA and IIB) within Lineage II, and divergent from those from the Americas (IIC). The two Vietnamese strains in IIA grouped together to the exclusion of the other Asian strain and the same grouping of Vietnamese strains was also seen in IIB. Furthermore, IIC including one Mexican strain (cheese_Mexico) and many North American strains defined an Americas clade and were separated from the Asian clades within Lineage II. However, this structure was imperfect with pig_ear_CA located in IIA, an otherwise Asian clade. The U.S. strains from various sources were diverse and grouped into both major lineages. All strains in Lineage III were isolated from the United States. *S*. Newport Lineages II and III were polyphyletic, namely, Lineage III displayed closer evolutionary relationship with *S*. Hadar and *S*. Typhimurium outgroups than Lineage II (Figure 1).

**Figure 2:**
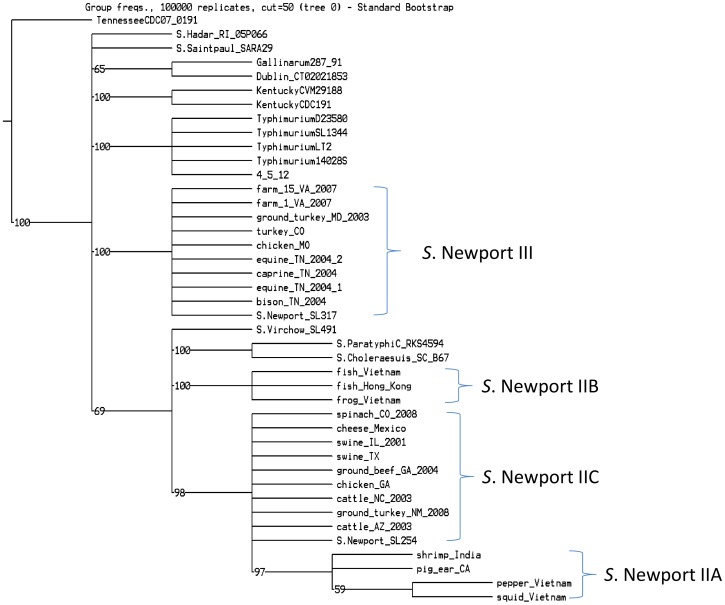
MLST analysis of *S*. Newport and outgroup genomes. Seven housekeeping genes were selected and MLST dendrogram was performed by TNT [38] with 100,000 iterations. *S*. Newport was divided into two major clusters, which were separated by outgroup genomes. Lineage II was divided into three sublineages, which display minor differences compared with the parsimony tree. Sublineage IIA showed closer relatedness with IIC than IIB.

Since multilocus sequence typing (MLST) has been used as a common analysis tool to study the phylogenetic relatedness and epidemiology of *Salmonella*, we extracted seven housekeeping genes (*aroC*, *dnaN*, *hemD*, *hisD*, *purE*, *sucA*, and *thrA*) of genomes in the current study (except strain from canine_AZ_2003 because of sequence quality) and performed MLST analyses (Figure 2). MLST indicated that *S*. Newport Lineage II was grouped into three sublineages with minor differences. For example, sublineage IIA showed closer relatedness with IIB than IIC. Additionally, Lineages II and III were separated by outgroup genomes, although outgroups displayed different relatedness compared with the parsimony phylogenetic tree (Figure 1). For example, *S*. Virchow, *S*. Paratyphi C and *S*. Choleraesuis showed closer relationship with Lineage II.

Furthermore, we listed pairwise SNP variation among these four *S*. Newport sublineages (IIA, IIB, IIC and III) and five different outgroups (Table 2). The inter-lineage SNP diversity was remarkable indicating the extensive genomic diversity within *S*. Newport. For example, the distance between Lineage III and sublineage IIB was approximately 36,800 SNPs, which was greater than that between Lineage III and *S*. Hadar RI_05P066 (approximately 34,400). Within Lineage II, sublineage IIC had closer relationship with IIB than IIA (Figure 1, Table 2).

**Table 2:** Average pairwise distance (no. of nucleotide difference) for the major groups shown in Figure 1.

	sublineage III	Hadar	Typhimurium	sublineage IIC	sublineage IIA	sublineage IIB	Dublin	Gallinarum
sublineage III								
Hadar	34418 (93)							
Typhimurium	36094 (90)	35900 (105)						
sublineage IIC	35048 (128)	37133 (147)	38640 (144)					
sublineage IIA	35627 (108)	37320 (152)	38893 (154)	17497 (95)				
sublineage IIB	36812 (106)	38529 (122)	38752 (131)	15605 (91)	25768 (85)			
Dublin	39879 (118)	40575 (133)	39275 (154)	40314 (175)	40878 (130)	41749 (136)		
Gallinarum	43027 (100)	43758 (159)	42824 (151)	43453 (158)	42666 (133)	44822 (124)	22070 (144)	
Kentucky	49260 (106)	49409 (80)	48612 (90)	50194 (96)	50694 (128)	48236 (146)	50955 (98)	53464 (96)

The valve refers to number of SNPs differences (standard deviation) between different selected groups and strains. The numbers of base differences per sequence from averaging over all sequence pairs between groups were shown.

We analyzed informative SNPs that define sublineages in the phylogenetic trees (Table S1). The SNPs that delineated the sublineages originated from various regions around the genome of *S*. Newport and included a variety of genes assigned to diverse functions including virulence, DNA replication and repair, and metabolism. For example, there were approximately 13,000 informative SNPs that changed only once and could differentiated Lineages II and III. Additionally, we analyzed genes defining the sublineages of Lineage II as well. For example, there are 2831, 2508, 1259 informative SNPs that defined sublineages IIA, IIB, IIC, respectively.

Moreover, we listed variable genes delineating various sublineages with their SNP changes, gene names and genome locus alignment coordinates (Table S1). We selected informative SNPs from 20 most variable genes (with highest number of informative SNPs that changed once and defined all members of each major lineage and sublineage) defining the two major lineages and delineating sublineages IIA, IIB and IIC. For example, *tpiA* gene (SNSL254_A4410) could be used as a marker to differentiate Lineages II and III. At position 91 of the alignment, nucleotides in *S*. Newport SL254 and SL317 were A and G, respectively, and amino acid changed from threonine to alanine. Variable genes found within sublineages IIA and IIB could be used as markers for Asian strains. Furthermore, the most variable genes with the largest numbers of informative SNPs could be used as targets of resequencing (Table S1). For example, there were 78 SNPs in *carB* (3228 bp) and 71 SNPs in *aceE* (2664 bp).

A cluster of multidrug-resistant *S*. Newport strains was placed in IIC, namely, node M in the parsimony tree (Figure 1). Previous studies indicated that *S*. Newport MDR-AmpC (resistant to third generation cephalosporins containing an ampC ß–lactamase gene [43], [44]) strains belonged to *S*. Newport Lineage II [14]. We identified the 20 most variable genes containing informative SNPs defining this sublineage (Table S1). Interestingly, *acrD* (SNSL254_A2674), encoding a multidrug efflux protein, contained 12 informative SNPs defining sublineage IIC with 3114 bp length. All strains of sublineage IIC had nucleotide C at the position 84, whereas all other *S*. Newport strains had T at the same locus.

Additionally, five MDR strains were grouped together, including cattle_AZ_2003, *S*. Newport SL254, cattle_NC_2003, cannine_AZ_2003, ground_beef_GA_2004 (Figure 1, Table 1). The one exception was swine_IL_2001, which was MDR but separated from the other MDR strains by ground_turkey_NM_2008 that was only resistant to Tetracycline. Furthermore, we analyzed the informative SNPs that delineating the five-MDR-strains group (Node M, Figure 1) within sublineage IIC and discovered a total of 33 informative SNPs (Table S1). It is notable that 17 of the informative SNPs were non-synonymous. For example, within *ksgA* gene encoding RNA dimethyltransferase associated with antibiotic resistance [45], the MDR group had nucleotide A at the position of 689, whereas the other *S*. Newport strains had G at the same position (Table S1), resulting in an amino acid change from asparagine to glycine.

We compared the genomic organization of *S*. Newport genomes of distinct subgroups (Figure S1). We used *S*. Newport SL254 as reference genome, which was completed genome and located in *S*. Newport Lineage IIC, to compare with *S*. Newport SL317, pig_ear_CA and fish_Vietnam, which located at Lineage III, IIA and IIB, respectively. Our MAUVE [36] data indicated that large indels and rearrangement events could be found, although the general genomic organizations are same.

Because of the importance of recombination events in the evolution of *S*. Newport, we performed ClonalFrame [40] analyses to display the effects of recombination events on the evolutionary history of *S*. Newport (Figure S2). Our data indicated that the r/m (Figure S2A: r/m equals the ratio of possibilities that a given site is altered through recombination event and substitution) and ρ/θ (Figure S2B: ρ/θ equals the ratio of rates of recombination event and substitution occur at a locus) ratios were 1.68 and 0.1, respectively. Moreover, the genomic representation mode was selected to display the recombination events (red line) and substitution (green triangle) on the node of II vs. III and IIA vs. IIB&C (Figure S2 C and D). Our data indicated that recombination events with Lineage II happened more frequently than the ones between Lineages II and III.

### Loci between *invH* and *mutS*

Genes at loci between *invH* and *mutS* genes in both Lineages II and III displayed distinct contents and were defined as Gene Clusters 1 and 2, respectively (Table 3). Because they were conserved within each lineage, *S*. Newport strains SL254 and chicken_MO were selected for further analysis of these gene clusters. All 15 outgroup genomes shared Gene Cluster 1 at the same locus with *S*. Newport SL254, although minor differences were found.

**Table 3:** Characteristics of genes/open reading frames (ORFs) between *invH* and *mutS* genes in Gene Cluster 1 of *S*. Newport SL254 and Gene Cluster 2 of strain from chicken_MO.

ORF	Gene Name	Size (bps)		GC%	Best Blastp Hit				Super Family
					Description	Source	E Value	Locus Tag	
**Gene Cluster 1 in ** ***S*** **. Newport SL254**									
A3107		282		49.3	Putative ABC-type transport system	*S*. Typhi CT18	2e-45	NP_457295.1	DUF1778
A3108		528		47.2	Acetyltransferase, gnat family	*S*. Typhi CT18	7e-99	NP_457296.1	NA
A3109	*tnp*	438		55.9	Transposase	*Enterobacter* *cloacae*	1e-81	AAV66983.1	NA
A3110	*pphB*	657		41.7	Serine/threonine-specific proteinphosphatase 2	*S*. TyphimuriumLT2	1e-125	AAL21787.1	MPP
A3111		495		48.7	Membrane protein	*S*. DublinCT_02021853	7e-92	ACH74700.1	NA
A3112		669		54.1	Hypothetical protein	*S*. SaintpaulSARA29	1e-125	EDZ12689.1	NA
**Gene Cluster 2 in strain from chicken_MO**									
11075	*insF*	738	52.3		Transposase InsF for insertionsequence IS3A/B/C/D/E/fA	*S*. Newport SL317	0	EDX51569.1	rve
11080	*insF*	171	55		Transposase InsF for insertionsequence IS3A/B/C/D/E/fA	*S*. Newport SL317	0	EDX51569.1	rve
11085		402	50.5		ISPsy11, transposase OrfA	*S*. Newport SL317	2e-92	EDX52090.1	NA
11090	*yis*	684	50.1		Integrase, catalytic region(ISPsy11, transposase OrfB)	*S*. Newport SL317	2e-170	EDX51974.1	rve
11095		258	47.3		ISEhe3 OrfA	*S*. Newport SL317	9e-57	EDX52144.1	HTH_Hin
11100	*tnpA*	90	46.7		Hypothetical protein	*S. Dublin*CT_02021853	6e-21	ACH75076.1	NA

Differences between Gene Cluster 1 and 2 demonstrated the mosaic genomic structure around *mutS* gene. Transposase and integrase were found in both sequences, indicating that both of them could be the hot spots for recombination events. The genes in both *S*. Newport SL254 and strain from chicken_MO are ordered top to bottom as their synteny on bacterial chromosome from 5′ to 3′.

In Gene Cluster 1 of *S*. Newport SL254, there were six genes that ranged from 282 to 669 bp, encoding ABC transport system protein, transposase, phosphatase and membrane protein (Table 3). They had distinct G+C% contents ranging from 41.7 to 55.9%. The best hits of blastp against these genes were found in various serotypes. For example, the best blast match of *pphB* gene in Gene Cluster 1 was *S.* Typhimurium LT2; and the best blastp match of *tnp* gene encoding a transposase in *S*. Newport SL254 was *Enterobacter cloacae* with 84% identities and 95% coverage. *S*. Typhimurium and *S*. Bardo had 62% identities and 95% coverage of *tnp* with *S*. Newport SL254. BLAST matches of other genes in Gene Cluster 1 were distributed broadly across *Salmonella* serotypes. Additionally, one large insertion (Gene Cluster 3) was observed at the 3′ end of Gene Cluster 1 in *S.* Newport strain from fish_Hong_Kong (Figure 1), including ORFs homologous to genes encoding transposase, integrase, phage related proteins and proteins of Type I Restriction Modification System in *Vibrio*. For example, according to blastp search *hsdS* gene showed 61% identities and 79% positives to gene in *Vibrio splendidus*; *hsdM* gene showed 84% identities and 92% positives to gene in *Vibrio metschnikovii*. Detailed information of Gene Cluster 3 was available in Table S2. These findings suggested that loci between *invH* and *mutS* were hot spots for horizontal gene transfer or recombination events and could facilitate acquisitions of new genetic elements.

A total of six genes ranging from 90 to 738 bp (46.7 to 55% G+C% contents) were identified in Gene Cluster 2 of chicken_MO (Table 3). The best blastp hits of the genes, except *tnpA* gene, were *S*. Newport SL317; and the best BLAST match of *tnpA* was *S*. Dublin CT_02021853. The best blastp hit of *insF* in chicken_MO was a transposase of *S*. Newport SL317 with 100% identities and 100% coverage, and *insF* was also found in *S.* arizonae 62:z4,z23 with 88% identities and 68% coverage and in *S*. Hadar RI_05P066 with 93% identities and 100% coverage, but not in other *Salmonella* serotypes. The other four genes in Gene Cluster 2 had a broader distribution among different serotypes of *Salmonella*. The best blastp hits of these four genes were proteins from *S*. Newport SL317 and *S*. Dublin CT_02021853 with at least 93% identities and with 100% coverage. Additionally, *tnpA* gene in chicken_MO was absent in *S*. Newport Lineage II and *S*. Virchow SL491 but present in all other genomes in the current study.

### Gene Cluster Encoding Fimbrial Operon and *cas* Genes

Similar to those between *invH* and *mutS*, genes at the 3′ end of *mutS* displayed significant variations between Lineages II and III, and genes of Lineage II shared high similarity with those of the outgroup genomes (Table 4). *steABCDEF* fimbrial operon located between *relA* and *mazG* genes was conserved in Lineage II and all outgroup genomes, but was not found in Lineage III. Blastp results demonstrated that this fimbrial operon was present in certain *Salmonella* serotypes. Lineage III strains had only two genes at the same locus, encoding RelE/ParE family plasmid stabilization system protein (SNSL317_A4074) and putative addiction module antidote protein (SNSL317_A4073). Interestingly, *steF* gene in *S*. Newport SL254 and genes between *relA* and *mazG* in *S*. Newport SL317 were found adjoining each other in *S*. Typhi CT18.

**Table 4:** Characteristics of genes/open reading frames (ORFs) between *relA* and *mazG* genes of *S*. Newport SL254 and SL317.

ORF	Gene Name	Size (bps)	GC%	Best Blastp Hit				Super Family
				Description	Source	E Value	Locus Tag	
***S*** **. Newport SL254**								
A3171	*steA*	588	49.3	putative fimbrial subunit	*S*. Newport SL254	3e-136	ACF63661.1	Fimbrial
A3172	*steB*	2646	55.5	fimbrial usher protein	*S*. Newport SL254	0	ACF64468.1	PRK15223
A3173	*steC*	774	55	chaperone protein PapD	*S*. Newport SL254	0	ACF62389.1	Pili_assembly
A3174	*steD*	507	56.6	fimbrial subunit	*S*. Newport SL254	2e-118	ACF63171.1	Fimbrial
A3175	*steE*	471	50.7	fimbrial subunit	*S*. Newport SL254	1e-110	ACF62527.1	Fimbrial
A3176	*steF*	537	52.3	fimbrial subunit	*S*. Newport SL254	1e-128	ACF62131.1	Fimbrial
***S*** **. Newport SL317**								
A4073		288	48.3	putative addiction moduleantidote protein	*S*. Typhi CT18	2e-47	NP_457351.1	RHH_2
A4074		297	38.7	plasmid stabilizationsystem protein, RelE/ParE family	*S*. Newport SL317	2e-51	ZP_02697812.2	Plasmid_stabil

We listed the detailed information of genes between *relA* and *mazG* genes. *S*. Newport SL254 and SL317 were selected. Our data indicated the genomic diversity of this region between Lineages II and III. Interestingly, ORF SNSL254_A3176 and SNSL317_A4073 were found adjoining together in *S*. Typhi CT18. The existence of *ste* fimbrial operon might enable Lineage II strains to infect variable hosts. The genes in both *S*. Newport SL254 and SL317 are ordered top to bottom as their synteny on bacterial chromosome from 5′ to 3′.

**Figure 3:**
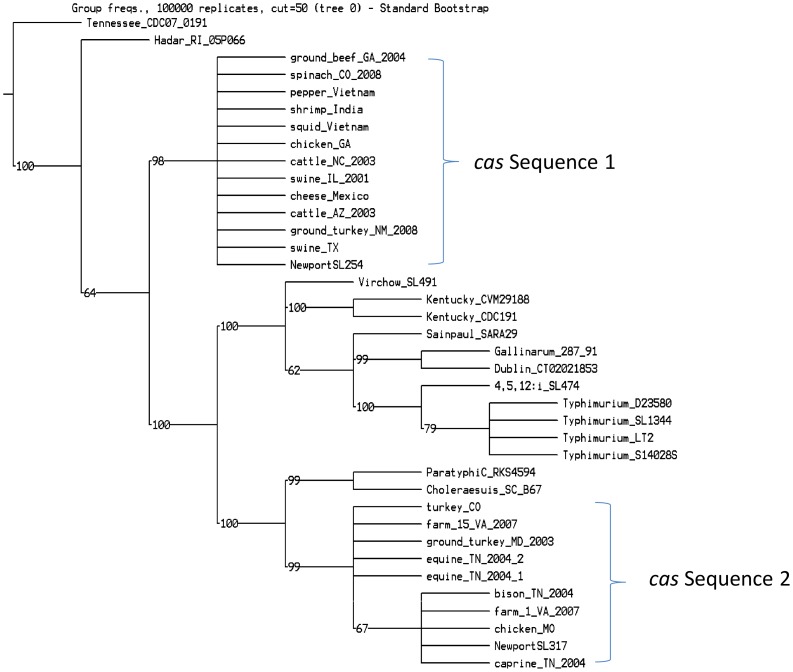
Parsimony phylogenetic tree for *cas* genes. We constructed this parsimony tree with 100,000 iterations by TNT [38] based on concatenated sequences of the *cas* genes. This dendrogram indicated that *cas* genes of Lineages II and III were originated from distinct sources.

We defined *cas* genes located at the 3′ end of *mutS* in Lineages II and III as *cas* Sequence 1 and 2, respectively. Multiple sequence alignments of concatenated nucleotide acids for *cas* Sequence 1 and 2 showed significant variations. Moreover, there are four collapsing groups in the parsimony tree because the sequence identities were almost 100% in each group, respectively. For example, there was only one substitution found at position 333 of strain from ground_turkey_MD_2003 in total 5,781 bp compared with other four sequences in the group (data not shown). A parsimony tree was generated based on *cas* alignments using TNT [38] with 100,000 bootstrap iterations (Figure 3), showing that *cas* proteins in Lineages II and III displayed divergent phylogenetic relatedness, and were separated by outgroup genomes. For example, *cas* genes of *S*. Paratyphi C and *S*. Choleraesuis displayed closer relatedness with *cas* Sequence 2 of Lineage III than Lineage II strains (Figure 3).

## Discussion

In the current study, whole genome sequencing data revealed that strains of *S*. Newport Lineages II and III are polyphyletic to each other and are separated by other *Salmonella* serotypes, including *S*. Hadar and *S*. Typhimurium (Figure 1). A phylogenetic tree based on whole genome sequencing data by Fricke et al. [15] suggested similar relationships and illustrated that *S.* Virchow SL491 phylogenetically displayed closer relationship with *S*. Newport lineages than others. In Fricke’s [15] study, 28 sequenced genomes representing 21 serotypes of *S. enterica* were selected to demonstrate the evolutionary history of sublineages of *S. enterica*. Conversely, we focused on variability between major lineages and sublineages of *S*. Newport with 15 outgroup genomes. Although a phylogenetic tree based on whole genome sequencing data provided a more accurate dendrogram than traditional subtyping methods [32], sampling was a critical factor to accomplish study research goals. Importantly, our data provides an insightful picture to reconstruct the evolutionary history of variation within *S*. Newport. As more sequenced *Salmonella* genomes become available, more accurate and comprehensive phylogenetic information will give us a better understanding of the evolution and ecology of *Salmonella*, for both subspecies and single serotypes [46].

**Figure 4:**
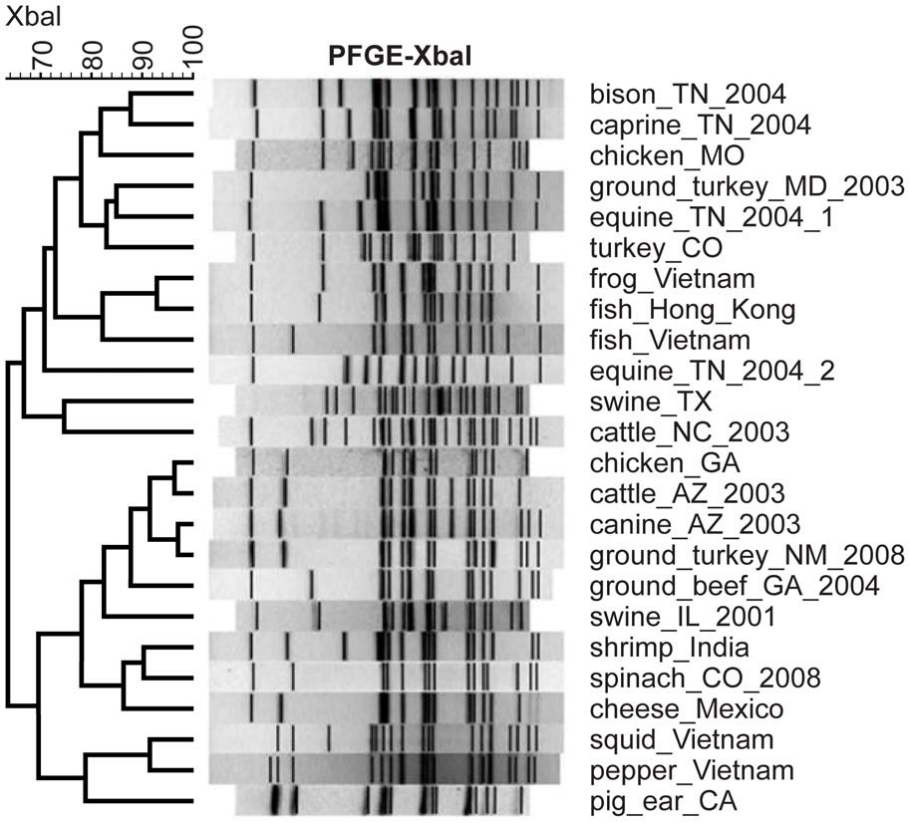
Pulsed Field Gel Electrophoresis (PFGE) profile digested with *Xba*I. We performed PFGE analysis of 24 *S*. Newport strains (without two environmental farm isolates) isolated from diverse sources and geographic locations. PFGE profiles divided these strains into two major clusters with different groupings compared with the phylogenetic tree based on whole genome wide SNPs.

Conventional subtyping methods, such as MLST and Pulsed Field Gel Electrophoresis (PFGE), have been used to differentiate pathogenic strains during outbreaks and trace-back investigations and to study the phylogenetic organization of pathogens. MLST analyses (Figure 2) indicated that *S*. Newport was divided into two major clusters and was separated by outgroup genomes. Lineage II was grouped into three sublineages; however, sublineage IIA displayed closer relatedness with IIC than IIB, which was different with the genomic based parsimony tree (Figure 1). This was not unexpected as the genomic database was significantly larger than the MLST database. MLST indicated that these seven housekeeping genes were valuable to differentiate major and sublineages of *S*. Newport, though MLST may not accurately show the relationships among the sublineages. Therefore, whole genome sequencing data was able to provide more accurate phylogenetic relationship than small sets of genes. However, PFGE often may not be able to differentiate highly clonal strains [28], [32]. A combination of whole genome sequencing and phylogenetic analysis has been proven to provide enough accuracy and sensitivity for epidemiological investigations [28], [32]. In the current study, comparisons of PFGE (Figure 4) and the phylogenetic tree (Figure 1) illustrated that PFGE was not able to delineate the major lineages correctly, as expected. For example, according to the PFGE profile (Figure 4), three strains of sublineage IIB, namely, frog_Vietnam, fish_Hong_Kong and fish_Vietnam, were located with strains of Lineage III to form a lineage unsupported by sequence analysis. Bell et al. [9] demonstrated that whole genome sequencing and phylogenetic analysis were able to differentiate *S*. Newport strains with an identical PFGE pattern during an outbreak case study, providing detailed information about *S*. Newport’s complex ecology to the investigators.

The parsimony tree had a clear geographic structure, which appears to be a common characteristic of *Salmonella* [28], [32]. *S.* Newport strains isolated from Asia were grouped together and divergent from those isolated from the Americas (Figure 1). Lineages II and III displayed extensive genomic diversity. For example, Lineage II strains from North America had closer evolutionary relatedness with those of Lineage II from Asia than ones of Lineage III from North America, suggesting that the geographic structure could be observed only among highly clonal lineages, but may not be apparent among the major lineages. Moreover, there was a diverse phylogenetic structure with *S*. Newport strain from cheese_Mexico genetically unique to other strains of North America (sublineage IIC in Figure 1), suggesting that *S*. Newport strains isolated from different states in the United States and in the Americas may have finer geographic structure. *S*. Newport strains from Asia showed very diverse geographic structure. Strains from Vietnam within sublineages IIA and IIB displayed closer relationship than with another Asian strain within the same sublineage (Figure 1). In addition, strains from Vietnam originated from different sublineages of Lineage II, such as frog_Vietnam and squid_Vietnam. However, there was one exception in that one isolate from the United States, pig_ear_CA was located within sublineage IIA, which was otherwise composed of Asian strains (Figure 1). This result indicated that *S*. Newport strains from Asia or this sublineage may have extensive genomic diversity and that geographic structure may be better identified among the most highly clonal lineages. We hypothesized that strain pig_ear_CA may be related to a food import or export from the Pacific Rim. Analysis of more isolates is needed to confirm the pig-ear sublineage.

The SNPs that delineating each sublineage (Table S1) were the most valuable for both targeted resequencing efforts and for rapid subtyping methods for trace-back of future *S*. Newport outbreak investigations and diagnosis, including the SNPs defining MDR strains, though plasmids likely play a critical role for antibiotic resistance of *S*. Newport [47], [48]. Sangal et al. [14] indicated that most of Lineage III strains were pan-susceptible and all MDR-AmpC strains were exclusively associated with two sequence types (STs) of Lineage II. Similarly, all MDR strains in the present study were grouped together within sublineage IIC. We hypothesized that the plasmids of MDR strains in the present study had the same backbone as *Y. pestis* plP1202 and *S*. Newport pSN254, which could be broadly disseminated among MDR pathogens via horizontal or vertical gene transfer [47]. Our data found that genes associated with antibiotic resistance, *acrD* and *ksgA*, could delineate sublineage IIC and node M (Figure 1), respectively. Matsumura et al. [49] suggested that *acrD* contributed significantly to the formation of biofilm of *E coli* K-12. AcrD also played a major role in the intrinsic and elevated resistance of *S.* Typhimurium to a wide range of compounds [50]. Lama et al. [51] indicated that a nonsense mutation of *ksgA* caused resistance to amicoumacin A in methicillin-resistant *Staphylococcus aureus* (MRSA).

The region around *mutS* was thought to be an old region in the genome because it was part of the DNA mismatch repair system and SPI-1, which was acquired after *Salmonella* and *E. coli* separated from their common ancestor 100 million years ago [52]. Our findings indicated that diversities around conserved regions of the genome were found and provided an insightful understanding of the evolutionary process. Loci around *mutS* were thought to be hot spots for horizontal gene transfer and recombination events because this region was associated with pathogenicity and positive selection [53], [54], [55]. For example, our data showed that Gene Cluster 1 between *invH* and *mutS* included genes encoding ABC transport system protein. Another recent study hypothesized that an ABC transporter gene was associated with the ability of *Salmonella* to acquire nutrients for survival during host infection [56] and drug resistance [57]. A major finding from our study is that Gene Cluster 1 exists in Lineage II and all outgroup genomes, but not in Lineage III, suggesting that strains from these two major lineages may have different pathogenic capability. Moreover, the existence of transposable elements also could facilitate further genetic exchange within Gene Cluster 1. For example, Gene Cluster 3 in strain from fish_Hong_Kong (Table S2) was inserted at the 3′ end of Gene Cluster 1, illustrating that the evolution of this region is an ongoing process. Additionally, our data suggested that genes encoding restriction-modification (RM) subunits of *Vibrio* were homologous to ORFs in Gene Cluster 3.

The limit of serological classification is that some unrelated strains were considered to be the same serotype [10], [58]. As more data is available, distinct lineages of the same serotype are commonly found [32]. Our data indicates that *S*. Newport displays extensive genomic variation between the two major lineages, which are separated by other serotypes. Our data shows approximately 13,000 informative SNPs differences (SNPs that change once and define all members of these two major lineages) between *S*. Newport Lineages II and III. Moreover, the pairwise distance matrix (Table 2) suggests that the number of SNP differences between Lineage III and any sublineage of Lineage II was larger than that between Lineage III and *S*. Hadar RI_05P066.

*S*. Newport has been proposed to be paraphyletic or polyphyletic [10], [11], [12], [13], [14] with distinct clonal lineages and it acted as a frequent donor or recipient of recombination events [46]. According to the cross-link analysis, Sangal et al. [14] hypothesized that Lineages II and III had arisen from a single lineage then differentiated or that recombination events frequently happened after Lineages II and III shared a niche and then would merge in the future. However, our data indicated that the recombination events between Lineages II and III were less frequent than those within Lineage II. In the current study, our phylogenetic analysis (Figure 1) demonstrated that Lineages II and III were polyphyletic and were divergent from each other by other *Salmonella* serotypes. The remarkable inter-lineage distance (Table 2) suggested that *S*. Newport Lineages II and III diverged early on in the serotype evolution of *S*. Newport and that they have evolved largely independently. Horizontal gene transfer and recombination events have been the major force for evolution of *S*. Newport [14] and our data supports that this pathogenic serotype has extensive genomic diversity. It is likely that geographic and ecological structure provided physical proximity to facilitate the recombination events among bacteria, which may form sublineages of pathogen populations [59].

Additionally, a study of the pan-genome family tree indicated that *S*. Newport SL254 was separated from *S*. Newport SL317 in some single gene trees by another serotype, *S*. Hadar RI_05P066 [34], which showed close relatedness with Lineage III in the present study (Figure 1, Table 2). This separation was confirmed by both the parsimony tree (Figure 1) and phylogenetic dendrogram of *cas* genes (Figure 3). In the current study, both of the MLST and *cas* genes could differentiate Lineage II and III; however, they could not delineate strains within Lineage II accurately. Therefore, full genome information or an improved MLST panel is needed to improve our understanding of the evolution of *Salmonella* [33], [46]. Lineages II and III may have acquired the *cas* gene cluster from various sources. Although we do not fully understand the process of this genetic exchange, horizontal gene transfer also occasionally happened to housekeeping genes and this supports the hypothesis that the loci around *mutS* are hot spots for horizontal gene transfer.

As shown in Table 4, *S*. Newport Lineage II and the outgroup genomes contained *ste* fimbrial operon between loci *relA* and *mazG* genes. The existence of the *ste* fimbrial operon may facilitate Lineage II strains differing in their adhesion abilities and competing within various ecological environments [33], [60]. As den Bakker et al. suggested [33], genes enriched in different bacterial subpopulations could reveal various selective pressures acting on different subpopulations. Because genes between loci *relA* and *mazG* of these two lineages were both adjoining in *S*. Typhi CT18, we thought this fimbrial operon may exist in Lineage III before it was lost. Thus, our data suggested that loci around *mutS* [61], [62] displayed mosaic structure because of recombination events.

## Supporting Information

Figure S1 **S1. Genomic organization comparisons between sublineages.**
(TIF)

Figure S2 **S2. ClonalFrame analyses of recombination events.**
(TIF)

Table S1 **S1. Most variable genes defining the major lineages and sublineages of** ***S*** **. Newport.**
(DOC)

Table S2 **S2. Characteristics of genes/open reading frames (ORFs) in Gene Cluster 3 of strain from fish_Hong_Kong.**
(DOC)
