# Supplementary figures and images for: Phylogenetics and Differentiation of Salmonella Newport Lineages by Whole Genome Sequencing
Source: PLoS One. 2013 Feb 11;8(2):e55687. doi: 10.1371/journal.pone.0055687 (PMC3569456; doi:10.1371/journal.pone.0055687)

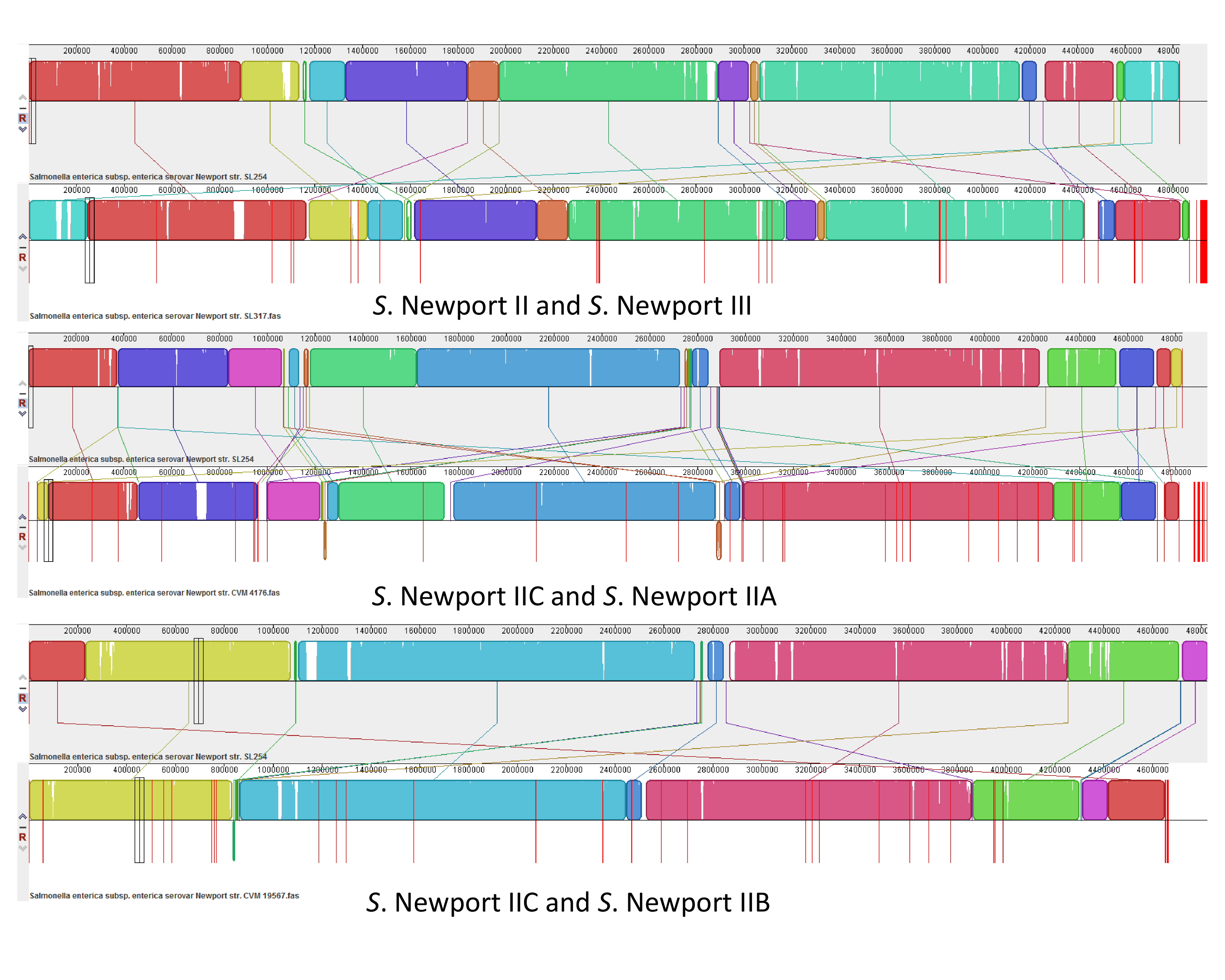

Supplement: Figure S1 — S1. Genomic organization comparisons between sublineages. (TIF) [file pone.0055687.s001.tif]

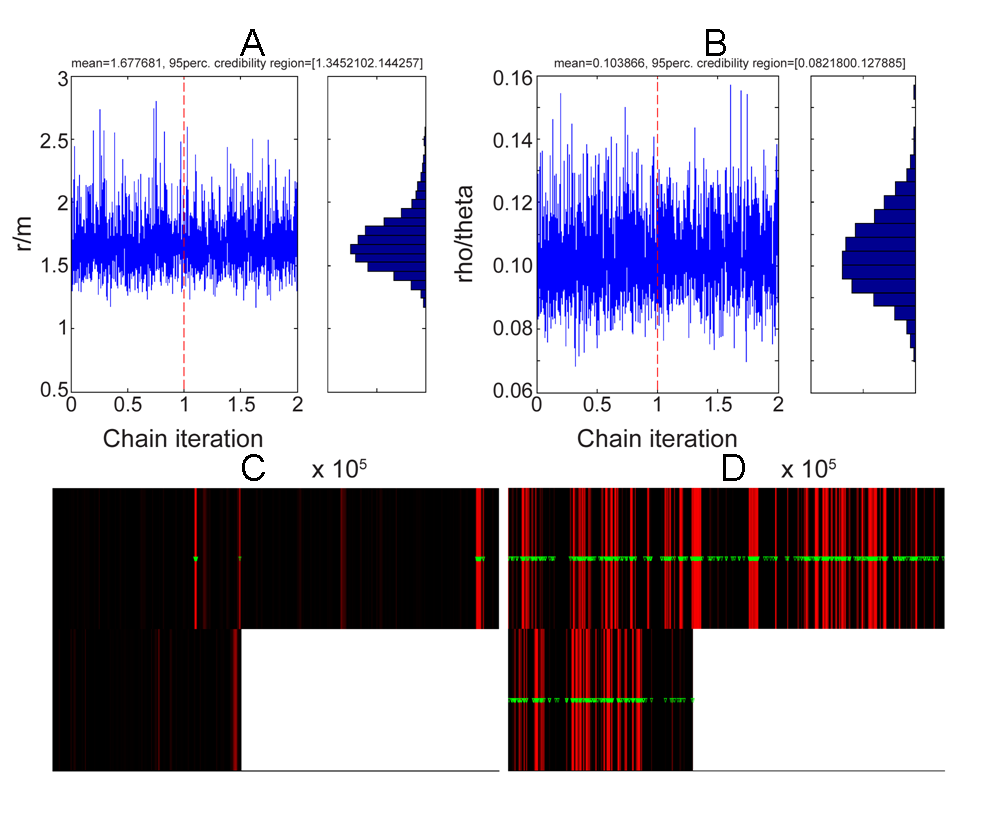

Supplement: Figure S2 — S2. ClonalFrame analyses of recombination events. (TIF) [file pone.0055687.s002.tif]
