# Supplementary material for: Phylogenetics and Differentiation of Salmonella Newport Lineages by Whole Genome Sequencing
Source: PLoS One. 2013 Feb 11;8(2):e55687. doi: 10.1371/journal.pone.0055687 (PMC3569456; doi:10.1371/journal.pone.0055687)
Supplement: Table S1 — S1. Most variable genes defining the major lineages and sublineages of S . Newport. (DOC) [file pone.0055687.s003.doc]

**Table S1. Most variable genes that defining major lineages and sublineages**

| Gene | Locus A | Locus B | Nuc | AA | Position | # of SNPs | Group | Description |
| --- | --- | --- | --- | --- | --- | --- | --- | --- |
| Genes define *S*. Newport Lineages II and III | | | | | | | | |
| *pduS* | SNSL254_A2230 | SNSL317_A3497 | G->A | A/T | 7 | 59 |  | polyhedral body protein |
| *thiC* | SNSL254_A4496 | SNSL317_A2323 | C->G | G | 72 | 59 |  | thiamine biosynthesis protein |
| *nrdA* | SNSL254_A2462 | SNSL317_A3257 | C->T | I | 186 | 51 |  | ribonucleoside-diphosphate reductase |
| *dmsA* | SNSL254_A4651 | SNSL317_A1619 | T->C | R | 315 | 51 |  | anaerobic dimethyl sulfoxide reductase |
| *ilvD* | SNSL254_A4186 | SNSL317_A2593 | G->A | L | 1275 | 47 |  | dihydroxy-acid dehydratase |
| *yacH* | SNSL254_A0171 | SNSL317_A1794 | T->G | S | 240 | 40 |  | putative outer membrane protein |
| *pduQ* | SNSL254_A2229 | SNSL317_A3498 | G->T | A | 111 | 38 |  | propanediol utilization: propanol dehydrogenase |
| *putA* | SNSL254_A1218 | SNSL317_A1473 | G->A | A | 873 | 38 |  | trifunctional transcriptional regulator |
| *rpoC* | SNSL254_A4487 | SNSL317_A2332 | C->T | L | 919 | 35 |  | DNA-directed RNA polymerase subunit |
| *gntR* | SNSL254_A3810 | SNSL317_A4927 | T->C | S | 255 | 34 |  | putative GntR-family regulatory protein |
| *hmt* | SNSL254_A0021 | SNSL317_A1949 | C->T | T/G | 1190 | 34 |  | putative hydroxymethyltransferase |
| *ytfN* | SNSL254_A4775 | SNSL317_A2173 | C->T | A/V | 59 | 33 |  | putative periplasmic protein |
| *norR* | SNSL254_A3041 | SNSL317_A4199 | G->T | V | 105 | 30 |  | anaerobic nitric oxide reductase transcription regulator |
| *yegQ* | SNSL254_A2321 | SNSL317_A3397 | T->C | G | 72 | 30 |  | peptidase U32 family protein |
| *tpiA* | SNSL254_A4410 | SNSL317_A2457 | A->G | T/A | 91 | 30 |  | triosephosphate isomerase |
| *araA* | SNSL254_A0109 | SNSL317_A1857 | T->C | I | 348 | 29 |  | L-arabinose isomerase |
| *hrpA* | SNSL254_A1758 | SNSL317_A0497 | C->T | L | 841 | 28 |  | ATP-dependent helicase |
| *hypF* | SNSL254_A3044 | SNSL317_A4196 | C->T | F | 264 | 28 |  | hydrogenase maturation protein |
| *infB* | SNSL254_A3544 | SNSL317_A3703 | C->T | G | 1026 | 27 |  | translation initiation factor IF-2 |
| *sucA* | SNSL254_A0795 | SNSL317_A0018 | A->G | E | 843 | 27 |  | 2-oxoglutarate dehydrogenase E1 component |
| Genes defines sublineage IIA | | | | | | | | |
| *recG* | SNSL254_A4024 | SEEN443_05060 | C->G | L | 858 | 36 | IIA | ATP-dependent DNA helicase |
| *parC* | SNSL254_A3430 | SEEN443_22651 | G->C | S/T | 170 | 30 | IIA | DNA topoisomerase IV subunit A |
| *nirC* | SNSL254_A3748 | SEEN443_16780 | G->T | A/S | 658 | 28 | IIA | nitrite transporter |
| *cysM* | SNSL254_A2634 | SEEN443_21787 | G->A | A | 480 | 25 | IIA | cysteine synthase B |
| *sgrR* | SNSL254_A4141 | SEEN443_14367 | C->T | P | 96 | 23 | IIA | HTH-type transcriptional regulator |
| *carB* | SNSL254_A0071 | SEEN443_13940 | T->C | C | 294 | 22 | IIA | carbamoyl phosphate synthase large subunit |
| *glmS* | SNSL254_A4142 | SEEN443_14372 | C->T | R | 222 | 20 | IIA | glucosamine--fructose-6-phosphateaminotransferase |
| *polB* | SNSL254_A0103 | SEEN443_13795 | C->T | G | 1080 | 20 | IIA | DNA polymerase II |
| *tktA* | SNSL254_A3322 | SEEN443_22146 | T->G | P | 294 | 19 | IIA | transketolase |
| *ligA* | SNSL254_A4019 | SEEN443_05035 | A->G | T/A | 103 | 19 | IIA | NAD-dependent DNA ligase |
| *pucJ* | SNSL254_A3907 | SEEN443_06684 | A->G | Q | 9 | 19 | IIA | xanthine permease |
| *cpdB* | SNSL254_A4766 | SEEN443_00595 | C->T | A | 780 | 18 | IIA | 3'-cyclic-nucleotide 2'-phosphodiesterase |
|  | SNSL254_A3270 | SEEN443_21941 | G->A | A/T | 55 | 18 | IIA | putative inner membrane protein |
| *levR* | SNSL254_A4052 | SEEN443_05190 | G->C | Q/H | 75 | 18 | IIA | sigma-54 dependent transcription regulator |
| *ybbP* | SNSL254_A0562 | SEEN443_07996 | G->A | L | 90 | 18 | IIA | efflux ABC transporter permease protein |
| *yggW* | SNSL254_A3351 | SEEN443_22286 | C->T | D | 183 | 17 | IIA | putative oxidase |
| *sgbU* | SNSL254_A3954 | SEEN443_06894 | C->A | P/T | 58 | 16 | IIA | putative L-xylulose 5-phosphate 3-epimerase |
| *phoU* | SNSL254_A4134 | SEEN443_14332 | C->T | A | 126 | 16 | IIA | phosphate transport system regulatory protein |
| *pepN* | SNSL254_A1098 | SEEN443_05597 | C->A | P/Q | 341 | 16 | IIA | aminopeptidase N |
| *metL* | SNSL254_A4432 | SEEN443_16720 | G->A | T | 816 | 16 | IIA | bifunctional aspartate kinase II |
| Genes define sublineage IIB | | | | | | | | |
| *hemL* | SNSL254_A0223 | SEEN447_13367 | G->C | T | 357 | 29 | IIB | glutamate-1-semialdehyde aminotransferase |
| *truB* | SNSL254_A3542 | SEEN447_18807 | C->T | T | 132 | 24 | IIB | tRNA pseudouridine synthase B |
| *gyrB* | SNSL254_A4120 | SEEN447_13058 | C->T | Y | 726 | 23 | IIB | DNA gyrase subunit B |
| *dho* | SNSL254_A4791 | SEEN447_14077 | C->T | H/Y | 199 | 22 | IIB | dihydroorotase |
| *purL* | SNSL254_A2768 | SEEN447_07165 | C->T | L | 175 | 22 | IIB | phosphoribosylformylglycinamidine synthase |
| *carA* | SNSL254_A0070 | SEEN447_20096 | G->A | E/T | 757 | 20 | IIB | carbamoyl phosphate synthase small subunit |
| *aceK* | SNSL254_A4522 | SEEN447_15307 | C->T | S | 741 | 19 | IIB | bifunctional isocitrate dehydrogenase kinase |
| *mgtA* | SNSL254_A4804 | SEEN447_14022 | C->A | R | 297 | 18 | IIB | magnesium-translocating P-type ATPase |
| *cyaA* | SNSL254_A4221 | SEEN447_11432 | C->T | L | 802 | 17 | IIB | adenylate cyclase |
| *uvrD* | SNSL254_A4231 | SEEN447_11487 | C->T | L | 1603 | 17 | IIB | DNA-dependent helicase II |
| *mtlA* | SNSL254_A3963 | SEEN447_08000 | A->T | S | 1222 | 16 | IIB | pts system mannitol-specific eiicba component |
| *creC* | SNSL254_A4947 | SEEN447_19716 | T->C | L | 199 | 14 | IIB | sensory histidine kinase |
| *glnS* | SNSL254_A0745 | SEEN447_07959 | T->C | Y | 576 | 14 | IIB | glutaminyl-tRNA synthetase |
| *trmE* | SNSL254_A4127 | SEEN447_13018 | C->T | R | 129 | 13 | IIB | tRNA modification GTPase |
| *mac* | SNSL254_A4603 | SEEN447_14967 | T->C | F/S | 65 | 13 | IIB | putative integral membrane protein |
| *yebU* | SNSL254_A1989 | SEEN447_10361 | T->C | Y/H | 7 | 13 | IIB | paral putative rRNA methyltransferase |
| *yniC* | SNSL254_A1436 | SEEN447_20726 | G->A | S/N | 203 | 12 | IIB | phosphatase |
| *treC* | SNSL254_A4800 | SEEN447_14037 | G->A | V/I | 1018 | 12 | IIB | alpha phosphotrehalase |
| *thiI* | SNSL254_A0472 | SEEN447_06040 | C->T | S | 1320 | 12 | IIB | thiamine biosynthesis protein |
| *barA* | SNSL254_A3180 | SEEN447_06891 | C->T | N | 1011 | 11 | IIB | hybrid sensory histidine kinase |
| Genes define sublineage IIC | | | | | | | | |
| *carB* | SNSL317_A1896 | SNSL254_A0071 | T->C | V | 132 | 36 | IIC | carbamoyl phosphate synthase large subunit |
| *yicL* | SNSL317_A4701 | SNSL254_A4029 | A->G | I/V | 100 | 26 | IIC | alpha-xylosidase |
| *ypfI* | SNSL317_A1096 | SNSL254_A2678 | A->C | R | 343 | 21 | IIC | acetyltransferase |
| *carA* | SNSL317_A1897 | SNSL254_A0070 | A->T | I | 60 | 18 | IIC | carbamoyl phosphate synthase small subunit |
| *yicH* | SNSL317_A4702 | SNSL254_A4028 | C->A | P | 453 | 17 | IIC | AsmA family protein |
| *speC* | SNSL317_A3890 | SNSL254_A3363 | T->G | V/G | 278 | 17 | IIC | ornithine decarboxylase isozyme |
| *guaA* | SNSL317_A1071 | SNSL254_A2703 | T->C | L | 346 | 15 | IIC | GMP synthase |
| *kdpD* | SNSL317_A0049 | SNSL254_A0764 | T->C | L | 1432 | 15 | IIC | sensor protein KdpD |
| *malP* | SNSL317_A4954 | SNSL254_A3788 | G->A | V/M | 1288 | 15 | IIC | maltodextrin phosphorylase |
| *malQ* | SNSL317_A4955 | SNSL254_A3787 | G->A | A/T | 646 | 15 | IIC | 4-alpha-glucanotransferase |
| *torS* | SNSL317_A4630 | SNSL254_A4109 | C->T | R/C | 97 | 14 | IIC | hybrid sensory histidine kinase |
| *cysW* | SNSL317_A1139 | SNSL254_A2636 | C->T | L | 367 | 13 | IIC | sulfate/thiosulfate transporter permease subunit |
| *sgbU* | SNSL317_A4778 | SNSL254_A3954 | C->G | Q/E | 271 | 13 | IIC | putative L-xylulose 5-phosphate 3-epimerase |
| *dbpA* | SNSL317_A0479 | SNSL254_A1775 | C->G | T/S | 203 | 13 | IIC | ATP-dependent RNA helicase |
| *acrD* | SNSL317_A1100 | SNSL254_A2674 | T->C | F | 84 | 12 | IIC | aminoglycoside/multidrug efflux system |
| *nrfC* | SNSL317_A1591 | SNSL254_A4624 | G->A | V/I | 52 | 12 | IIC | cytochrome c-type biogenesis protein |
| *guaB* | SNSL317_A1070 | SNSL254_A2704 | C->T | S | 411 | 12 | IIC | inosine-5'-monophosphate dehydrogenase |
| *torA* | SNSL317_A4634 | SNSL254_A4105 | T->C | G | 60 | 12 | IIC | trimethylamine-N-oxide reductase |
| *kdpB* | SNSL317_A0047 | SNSL254_A0766 | G->A | G/D | 200 | 11 | IIC | potassium-transporting ATPase subunit B |
| *malZ* | SNSL317_A2775 | SNSL254_A0446 | T->C | H | 258 | 10 | IIC | maltodextrin glucosidase |
| Genes define node M | | | | | | | | |
| *rfaD* | SNSL317_A4743 | SNSL254_A3990 | G->T | A/S | 928 | 1 | MDR | ADP-L-glycero-D-manno-heptose-6-epimerase |
|  | SNSL317_A0618 | SNSL254_A1638 | G->A | V | 222 | 1 | MDR | glutaminase |
|  | SNSL317_A2697 | SNSL254_A0373 | T->G | S/A | 892 | 1 | MDR | haloacetate dehalogenase H-1 |
| *fadH* | SNSL317_A3774 | SNSL254_A3480 | T->C | C | 1062 | 1 | MDR | FAD/FMN-binding/pyridine nucleotide-disulphide oxidoreductase family protein |
| *pstA* | SNSL317_A4598 | SNSL254_A4136 | C->T | I | 429 | 1 | MDR | phosphate transporter permease subunit |
| *yebZ* | SNSL317_A0217 | SNSL254_A2033 | G->A | V/I | 604 | 1 | MDR | copper resistance protein D |
| *ksgA* | SNSL317_A1870 | SNSL254_A0095 | G->A | G/D | 689 | 1 | MDR | dimethyladenosine transferase |
| *yedP* | SNSL317_A0096 | SNSL254_A2149 | G->A | R/H | 752 | 1 | MDR | mannosyl-3-phosphoglycerate phosphatase |
| *suhB* | SNSL317_A1027 | SNSL254_A2746 | G->C | G/R | 448 | 1 | MDR | inositol monophosphatase |
| *ydiY* | SNSL317_A0820 | SNSL254_A1441 | A->T | S | 165 | 1 | MDR | outer membrane protein |
| *hmt* | SNSL317_A1949 | SNSL254_A0021 | T->C | L | 2301 | 1 | MDR | putative hydroxymethyltransferase |
| *hycC* | SNSL317_A4186 | SNSL254_A3054 | T->G | L/V | 724 | 1 | MDR | formate hydrogenlyase subunit 3 |
| *proQ* | SNSL317_A0270 | SNSL254_A1985 | C->T | P/S | 259 | 1 | MDR | putative solute/DNA competence effector |
| *uvrY* | SNSL317_A0135 | SNSL254_A2110 | A->T | Q/L | 284 | 1 | MDR | response regulator |
| *argO* | SNSL317_A3941 | SNSL254_A3305 | A->G | A | 317 | 1 | MDR | arginine exporter protein |
| *atpI* | SNSL317_A4581 | SNSL254_A4153 | T->C | P | 153 | 1 | MDR | ATP synthase F0, I subunit |
| *nuoG* | SNSL317_A3209 | SNSL254_A2507 | C->T | T | 231 | 1 | MDR | NADH dehydrogenase subunit G |
| *tppB* | SNSL317_A0697 | SNSL254_A1562 | G->A | V | 612 | 1 | MDR | tripeptide transporter permease |
| *yhjJ* | SNSL317_A4850 | SNSL254_A3885 | G->A | A | 870 | 1 | MDR |  |
| *infB* | SNSL317_A3703 | SNSL254_A3544 | C->T | L | 307 | 1 | MDR | translation initiation factor IF-2 |
| *ptsG* | SNSL317_A4398 | SNSL254_A1302 | T->G | S/A | 43 | 1 | MDR | glucose-specific PTS system IIBC components |
| *ulaA* | SNSL317_A2201 | SNSL254_A4744 | C->T | T/I | 83 | 1 | MDR | ascorbate-specific PTS system enzyme IIC |
| *ybfM* | SNSL317_A0067 | SNSL254_A0746 | C->T | T | 1167 | 1 | MDR | outer membrane porin, OprD family |
| *mac* | SNSL317_A1571 | SNSL254_A4603 | C->T | Y | 21 | 1 | MDR | integral membrane protein |
| *ygdH* | SNSL317_A4058 | SNSL254_A3192 | C->T | C | 555 | 1 | MDR | lysine decarboxylase family protein |
| *ynfM* | SNSL317_A2693 | SNSL254_A0369 | T->G | F/V | 286 | 1 | MDR | permease |
| *adiA* | SNSL317_A1610 | SNSL254_A4642 | T->A | I | 459 | 1 | MDR | biodegradative arginine decarboxylase |
| *wzzE* | SNSL317_A2580 | SNSL254_A4199 | T->C | I/T | 314 | 1 | MDR | lipopolysaccharide biosynthesis protein |
| *aroH* | SNSL317_A0802 | SNSL254_A1458 | A->C | Q/P | 449 | 1 | MDR | phospho-2-dehydro-3-deoxyheptonate aldolase |
| *speC* | SNSL317_A3890 | SNSL254_A3363 | T->G | V/G | 980 | 1 | MDR | ornithine decarboxylase |
| *yrbG* | SNSL317_A3673 | SNSL254_A3575 | C->G | L/V | 772 | 1 | MDR | calcium/sodium:proton antiporter |
| *fcl* | SNSL317_A3437 | SNSL254_A2290 | G->A | V/M | 94 | 1 | MDR | GDP-L-fucose synthetase |
| *cobD* | SNSL317_A3518 | SNSL254_A2210 | C->A | G | 555 | 1 | MDR | cobalamin biosynthesis protein |
| The 20 most variable genes | | | | | | | | |
| *carB* | SNSL317_A1896 | SNSL254_A0071 |  |  |  | 78 |  | carbamoyl phosphate synthase large subunit |
| *aceE* | SNSL317_A1800 | SNSL254_A0165 |  |  |  | 71 |  | pyruvate dehydrogenase subunit E1 |
| *hrpA* | SNSL317_A0497 | SNSL254_A1758 |  |  |  | 69 |  | ATP-dependent RNA helicase |
| *putA* | SNSL317_A1473 | SNSL254_A1218 |  |  |  | 66 |  | trifunctional transcriptional regulator |
| *prpD* | SNSL317_A2737 | SNSL254_A0410 |  |  |  | 64 |  | 2-methylcitrate dehydratase |
| *acnB* | SNSL317_A1793 | SNSL254_A0172 |  |  |  | 62 |  | bifunctional aconitate hydratase 2 |
| *opdA* | SNSL317_A4871 | SNSL254_A3864 |  |  |  | 60 |  | oligopeptidase A |
| *thiC* | SNSL317_A2323 | SNSL254_A4496 |  |  |  | 59 |  | thiamine biosynthesis protein |
| *pduS* | SNSL317_A3497 | SNSL254_A2230 |  |  |  | 59 |  | polyhedral body protein |
| *dho* | SNSL317_A2157 | SNSL254_A4791 |  |  |  | 58 |  | dihydroorotase |
| *dmsA* | SNSL317_A1619 | SNSL254_A4651 |  |  |  | 55 |  | anaerobic dimethyl sulfoxide reductase chain A |
| *carA* | SNSL317_A1897 | SNSL254_A0070 |  |  |  | 54 |  | carbamoyl phosphate synthase small subunit |
| *thrA* | SNSL317_A1968 | SNSL254_A0002 |  |  |  | 54 |  | bifunctional aspartokinase I/homoserine dehydrogenase I |
| *yhiQ* | SNSL317_A4872 | SNSL254_A3863 |  |  |  | 53 |  | methyltransferase |
| *fadH* | SNSL317_A3774 | SNSL254_A3480 |  |  |  | 53 |  | FAD/FMN-binding/pyridine nucleotide-disulfide oxidoreductase family protein |
| *recG* | SNSL317_A4706 | SNSL254_A4024 |  |  |  | 53 |  | ATP-dependent DNA helicase |
| *yicJ* | SNSL317_A2440 | SNSL254_A4394 |  |  |  | 53 |  | sugar (Glycoside-Pentoside-Hexuronide) transporter |
| *yacH* | SNSL317_A1794 | SNSL254_A0171 |  |  |  | 52 |  | putative outer membrane protein |
| *nrdA* | SNSL317_A3257 | SNSL254_A2462 |  |  |  | 52 |  | ribonucleotide-diphosphate reductase subunit alpha |
| *ypfI* | SNSL317_A1096 | SNSL254_A2678 |  |  |  | 51 |  | acetyltransferase |

Variable genes were listed by their GenBank abbreviation and function description and by the locus to *S*. Newport SL254, SL317 and two Asian strains (strains from shrimp_India and frog_Vietnam). A representative nucleotide change observed within each gene is listed as well as whether this caused an amino acid change and to which phylogenetic group it was associated with from Figure 1. These genes and SNPs were the most valuable for the targeted resequencing and rapid subtyping methods for outbreak investigations. We listed 20 most variable genes that defining major and sub lineages. Moreover, we listed 33 informative SNPs that defining the MDR clade. # of SNPs means that the SNPs that changing once and defining members of major and sublineages.
