# Supplementary material for: Phylogenetics and Differentiation of Salmonella Newport Lineages by Whole Genome Sequencing
Source: PLoS One. 2013 Feb 11;8(2):e55687. doi: 10.1371/journal.pone.0055687 (PMC3569456; doi:10.1371/journal.pone.0055687)
Supplement: Table S2 — S2. Characteristics of genes/open reading frames (ORFs) in Gene Cluster 3 of strain from fish_Hong_Kong. (DOC) [file pone.0055687.s004.doc]

**Table S2. Characteristics of genes/open reading frames (ORFs) in Gene Cluster 3 of strain from fish_Hong_Kong.**

| ORF | Gene Name | Size (bps) | GC% | Best Blastp Hit | | | | Super Family |
| --- | --- | --- | --- | --- | --- | --- | --- | --- |
|  |  |  |  | Description | Source | E Value | Locus Tag |  |
| 18800 | *fic* | 618 | 49 | FIC domain-containing protein (cell filamentation) | *Klebsiella pneumoniae* KCTC 2242 | 7e-129 | AEJ99567.1 | Fic |
| 18795 |  | 171 | 45.6 | hypothetical protein | *Klebsiella pneumoniae* KCTC 2242 | 1e-28 | AEJ99568.1 | NA |
| 18790 |  | 810 | 44.4 | hypothetical protein | *Acinetobacter baumannii* SDF | 8e-160 | CAP02803.1 | HNHc |
| 18785 |  | 2002 | 43.2 | hypothetical protein | *Shewanella* sp. ANA-3 | 0 | ABK49185.1 | P-loop NTPase |
| 18780 |  | 237 | 42.6 | hypothetical protein | *Acinetobacter* baumannii SDF | 7e-37 | CAP02818.1 | NA |
| 18775 | *hsdS* | 1230 | 38.6 | type I restriction enzyme specificity protein | *Vibrio splendidus*  12B01 | 0 | EAP93672.1 | Methylase_S |
| 18770 | *hsdM* | 1536 | 41.3 | type I restriction-modification system DNA-methyltransferase  subunit M | *Vibrio metschnikovii* CIP 69.14 | 0 | EEX37915.1 | HsdM_N;  AdoMet_MTases |
| 18765 |  | 1473 | 45.8 | hypothetical protein | *Yersinia intermedia* ATCC 29909 | 0 | EEQ20934.1 | NA |
| 05488 | *tnpR* | 126 | 47.6 | IS10 transposase | *S*. Kentucky | 1e-22 | ADK62113.1 | NA |
| 05493 |  | 453 | 45.3 | hypothetical protein | *Yersinia intermedia* ATCC 29909 | 2e-108 | EEQ20933.1 | NA |
| 05498 |  | 1692 | 44.3 | hypothetical protein | *Yersinia intermedia* ATCC 29909 | 0 | EEQ20932.1 | DUF927 |
| 05503 |  | 879 | 41.3 | hypothetical protein | *Yersinia intermedia* ATCC 29909 | 7e-137 | EEQ20931.1 | NA |
| 05508 |  | 528 | 49.6 | hypothetical protein | *Yersinia intermedia* ATCC 29909 | 6e-05 | EEQ20929.1 | NA |
| 05513 |  | 558 | 35.3 | acyltransferase | *Thermoanaerobacterium thermosaccharolyticum* DSM 571 | 6e-04 | NC_014410.1 | NA |
| 05518 | *int* | 930 | 57.6 | integrase/recombinase  (Phage related) | *E. coli* WV_060327 | 0 | EFW70420.1 | DNA_BRE_C |
| 05523 | *radC* | 453 | 53.9 | putative phage DNA repair protein | *E. coli* SE15 | 6e-106 | BAI54704.1 | MPN |
| 05528 |  | 470 | 53.4 | hypothetical protein | *E. coli* SE15 | 5e-111 | BAI54703.1 | NA |
| 05533 |  | 423 | 42.6 | hypothetical protein | *E. coli* SE15 | 8e-98 | BAI54702.1 | DUF2787 |
| 05538 |  | 489 | 50.5 | hypothetical phage protein | *E. coli* SE15 | 8e-107 | BAI54701.1 | NA |
| 05543 | *nlp* | 270 | 50.7 | phage DNA-binding protein | *E. coli* SE15 | 7e-57 | BAI54700.1 | Nlp |
| 05548 |  | 528 | 46.6 | hypothetical protein | *E. coli* SE15 | 4e-127 | BAI54699.1 | NA |
| 05553 |  | 360 | 49.7 | hypothetical protein | *E. coli* SE15 | 7e-74 | BAI54698.1 | NA |
| 05558 |  | 336 | 42.3 | hypothetical protein | *E. coli* WV_060327 | 4e-72 | EFW70428.1 | NA |
| 05563 |  | 1188 | 30.3 | hypothetical protein | *Pseudomonas fluorescens* WH6 | 6e-54 | EFQ66029.1 | NA |
| 05568 | *int* | 1287 | 47.1 | Phage integrase | *Yersinia intermedia* ATCC 29909 | 0 | EEQ20915.1 | DNA_BRE_C |

Our data revealed that loci around *mutS* gene are hot spots for recombination events and it is an ongoing evolutionary process. For example, Gene Cluster 3 was found adjoining with Gene Cluster 1 within strain from fish_Hong_Kong. This region consisted of genes associated with Type I Modification Restriction System, recombinase, transposase and bacteriophage. The genes in Gene Cluster 3 are ordered top to bottom as their synteny on bacterial chromosome from 5’ to 3’.
